# A Retrospective Study of the Epidemiology, Histomorphological Features, Hormonal Status, and Radiological Features of Breast Cancer Diagnosed on Biopsies in Women in Bahrain Up to the Age of 40: A Single-Center Study

**DOI:** 10.7759/cureus.49228

**Published:** 2023-11-22

**Authors:** Zainab A Toorani, Zainab F Harb, Fatima M Alalawi, Zain Alattar, Nusaiba B Alzayani, Kawthar A Alasmawi, Rola Husain, Maha E Alsendi

**Affiliations:** 1 Pathology, Salmaniya Medical Complex, Manama, BHR; 2 Pathology, Royal College of Surgeons in Ireland - Medical University of Bahrain, Muharraq, BHR; 3 Musculoskeletal Radiology, Salmaniya Medical Complex, Manama, BHR; 4 Oncology, Salmaniya Medical Complex, Manama, BHR

**Keywords:** breast histopathology, oncology, young females, breast cancer, epidemiology of breast cancer

## Abstract

Background: Breast cancer is the most commonly diagnosed cancer in women worldwide. There are many risk factors that contribute to breast cancer which involve modifiable and non-modifiable factors. Most of the patients diagnosed with breast cancer are over 50 years of age, with breast cancer in women less than 40 years of age being relatively rare and typically more aggressive variants. Moreover, radiological examination is essential for diagnosis and triaging patients for further diagnostic procedures including tissue biopsy sampling. Despite the rarity of malignancy among the younger age group, all of their breast lesions are usually biopsied. Hence, this paper outlines the percentage of benign and malignant breast lesions detected on biopsies obtained from female patients who presented to our hospital before or at the age of 40.

Methods: We conducted a single institution retrospective study on 267 breast biopsies done for female patients before or at the age of 40 in the period from January 2020 to January 2023. The data was obtained from the National Health Information System at Salmaniya Medical Complex. The data collected included clinical history, histological, and radiological findings. Data on prognostic markers (estrogen receptor (ER)/progesterone receptor (PR) and human epidermal growth factor receptor 2 (HER2) status) were also included. The distribution of samples was established according to age ranges, pathological diagnostic categories (B1-5), and prognostic marker interpretation. Further subdivision was performed on cases with malignancy according to tumor grade. The frequency distribution was obtained for ER, PR, and HER status jointly. The association between age and grade, as well as age and categories, was also determined. All the analyses were performed using SPSS Statistics version 26.0 (IBM Corp. Released 2019. IBM SPSS Statistics for Windows, Version 26.0. Armonk, NY: IBM Corp), and the statistical significance was tested at a 5% level.

Results: Out of 267 samples, the majority (62.9%) were in patients with a range of age between 30 and 40. There were 68.5% of samples with the B2 (benign) category, followed by 20.2% with the B5b (malignant-invasive carcinoma) category. Out of 61 malignant samples, there were 17 (6.4%) samples positive on ER and PR but negative on HER2, 16 (6.0%) samples negative on ER, PR, and HER2, eight (3.0%) samples negative on ER and PR negative but positive on HER2, and five (8.2%) samples positive on ER, PR, and HER2. The majority of malignant cases were of grade II which accounted for 29 (10.9%) samples, followed by 23 (8.6%) with grade III. The association between age and grade was statistically not significant (p=0.113). However, the association between age and B categories was statistically significant with a p-value of 0.0002. A significantly higher proportion of cases with B5a (malignant-in situ carcinoma) or B5b (malignant-invasive carcinoma) categories were in the age range of 31-35 years and 36-40 years.

Conclusion: Breast cancer is rare among younger women. It mostly occurs in women over the age of 40 years. In women under the age of 40, it usually presents as a self-detected palpable mass and can show various radiological findings in accordance with the histological grade. Ultrasonography is the main method for the diagnosis of breast cancer especially in younger women, whereas mammography and MRI can contribute to both diagnosis and assessment of the extent of the disease.

## Introduction

Breast diseases are one of the most common diseases that affect women, and those who present to the clinic often complain of the presence of a new lump. Breast masses have a wide differential, one of them being breast cancer, which is the most prevalent cancer in women. Benign breast diseases are more common than breast cancer occurring in 25% to 50% of women [1]. Taking a diagnostic biopsy is required to classify breast masses into certain histologic categories that are used to indicate different diagnoses, B1 being normal breast tissue, B2 meaning a benign lump, B3 is a group of lesions that may have potential malignant tendencies, B4 indicates a suspicious lesion, and B5 represents a malignant lump. In situ and invasive carcinomas are included in the B5a and B5b subcategories, respectively, of the B5 category [2]. There are two most common breast lumps that are benign in nature, and they are fibroadenomas and cysts, affecting women between the ages of 35 and 50, as well as women between the ages of 20 and 30 [3]. A recent study published in 2023 by Chaabna et al. outlined that between the years 1998 and 2012, the incidence of breast cancer among the Gulf Cooperation Council countries was highest in Bahrain which accounted for an age-standardized rate of 61.85 per 100,000 women followed by Kuwait, Qatar, the United Arab Emirates, and Oman, and the lowest rates were recorded in the Kingdom of Saudi Arabia (KSA). Breast cancer incidences are not equal worldwide. For example, a study about breast cancer incidence in the Gulf countries stated that from the years 2013 until 2016, incidences were only documented in Qatar and KSA, with Qatar having a higher incidence than KSA [4]. Another study recorded the global incidence and found it to be highest in Belgium (112.3 per 100,000) and lowest in Iran (41.0 per 100,000) [5]. In most cases, breast cancer is found in older women. However, breast cancer can occur in younger females due to certain risk factors, such as the inheritance of mutated BRCA1 or BRCA2 genes or the use of contraceptives [6]. Younger women are more prone to getting a more aggressive form of breast cancer, like triple-negative breast cancer (estrogen receptor (ER)/progesterone receptor (PR) negative and human epidermal growth factor receptor 2 (HER2) negative for amplification). In addition, the survivability rates are lower, and the prognosis is worse than for older women. Moreover, younger women also have a higher rate of recurrence with the possibility of becoming amenorrhoeic and infertile [6,7]. This article is an institutional study of the number of women who presented at a single center in Bahrain with a breast lump from the year 2020 until the year 2023. Our aim is to emphasize the ages of the females and the diagnoses of these masses using histological breast biopsies. This paper studies the breast biopsy categories, Breast Imaging Reporting and Data System (BI-RADS) categories, histological grade, hormone receptors, and HER2 receptors. The data was collected from Bahrain's main tertiary hospital, Salmaniya Medical Complex, using the National Health Information System database system (I-SEHA).

## Materials and methods

We conducted a single-institution retrospective study on 267 breast biopsies done for female patients at or before the age of 40 in the period from January 2020 to January 2023. The data was obtained from the I-SEHA at Salmaniya Medical Complex, Manama, Bahrain. The Research Committee for Government Hospitals approved the study (79090723). During this three-year period, a total of 270 samples were examined. Of those, 267 samples were eligible, according to the inclusion and exclusion criteria. The data collected included clinical history, histological, and radiographic findings. Data on pathologic diagnostic category, tumor grade, and prognostic markers (ER/PR and HER2 status) were all retrieved from the histopathology reports. The distribution of samples was established according to age ranges, pathological diagnostic categories (B1-5), and prognostic marker interpretation. Further subdivision was performed on cases with malignancy according to tumor grade. The frequency distribution was obtained for ER, PR, and HER2 status jointly. The association between age and grade, as well as age and categories, was also determined.

Inclusion criteria are all breast biopsies done for female patients in the ≤40 year age group in Salmaniya Medical Complex in the period from January 2020 to January 2023. Among these samples, three cases were excluded. These cases include one breast skin punch biopsy and two samples with inadequate reports. Breast needle core biopsies done for patients >40 years old were already excluded from the cohort.

All the cases that met the inclusion criteria were also reported in accordance with the Royal College of Pathologists (RCPath) dataset for reporting breast needle core biopsies using five major diagnostic categories (B1-5). In brief, the B1 category represents normal tissue or non-diagnostic biopsies, the B2 category is for benign lesions, the B3 category is for lesions with uncertain malignant potential, the B4 category is for lesions suspicious for malignancy, and the B5 category is for malignant lesions. The B5 category is further subdivided generally into B5a for in situ carcinoma and B5b for invasive carcinoma.

ER and PR as well as ERBB2 oncogene (also more commonly known as HER2) expression were all assessed using immunohistochemical (IHC) stains that were performed on formalin-fixed, paraffin-embedded sections of mainly malignant breast biopsies (category 5). ER and PR IHC stains are reported to be positive when at least 1% of tumor cells show nuclear immunoreactivity. HER2 IHC stains are reported in accordance with the American Society of Clinical Oncology (ASCO) guidelines which depend on the assessment of the staining strength, completeness of membranous staining, and percentage of tumor cells with positive staining. HER2 scoring includes scores 0 and 1 which are interpreted as negative, score 2 which is for equivocal cases, and score 3 for clear-cut positive cases. Cases with score 2 (equivocal) are usually followed up with further in situ hybridization testing for HER2 amplification, with HER2 status (amplified vs. non-amplified) reported, but the results of the amplification testing are not included in the study. Tumor grading is done on all invasive malignancies and basically depends on the assessment of tumor differentiation, nuclear atypia, and mitotic activity [1].

The distribution of samples was established according to age ranges and thus obtained frequencies and percentages. Therefore, a cross-table for the ER and PR status of samples was obtained, and the association between the two was determined using Pearson’s chi-square test. The frequency distribution was also obtained for ER, PR, and HER2 status jointly. The association between age and grade, as well as age and categories, was also determined using Pearson’s chi-square test. All the analyses were performed using SPSS Statistics version 26.0 (IBM Corp. Released 2019. IBM SPSS Statistics for Windows, Version 26.0. Armonk, NY: IBM Corp), and the statistical significance was tested at a 5% level.

## Results

**Figure 1:**
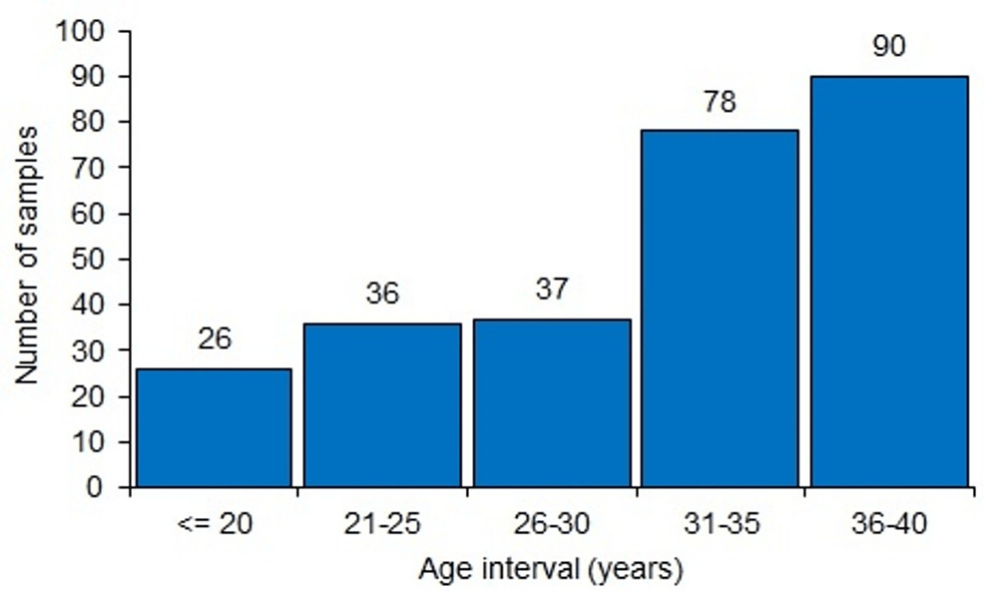
Histogram representation of the sample distribution in each age category

Both Table 1 and Figure 1 provide the distribution of samples according to age categories. Maximum samples (90, 33.7%) belonged to the age group 36-40 years, followed by 78 (29.2%) in the age group 31-35 years. The distribution was nearly equal in the range of 21-25 and 26-30 years. Figure 1 gives the histogram representation of the sample distribution.

**Table 1:** Distribution of samples according to age (years)

Age categories (years)	n	%
≤20	26	9.7
21-25	36	13.5
26-30	37	13.9
31-35	78	29.2
36-40	90	33.7
Total	267	100.0%

**Figure 2:**
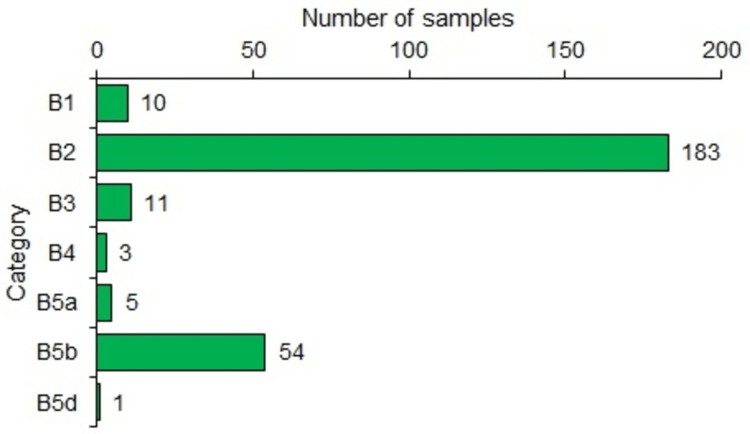
Horizontal bar chart showing the number of samples as per category B1: not diagnostic/normal tissue, B2: benign, B3: lesions with uncertain potential, B4: suspicious of malignancy, B5a: malignant-in situ carcinoma, B5b: malignant-invasive carcinoma, B5d: malignant tumor of divergent histology

Both Table 2 and Figure 2 provide the distribution of samples according to pathological diagnostic categories. Maximum samples (183, 68.5%) had the B2 category, followed by 54 (20.2%) with the B5b category. There were 11 (4.1%) samples with the B3 category and 10 (3.7%) samples with the B1 category.

**Table 2:** Distribution of samples according to pathological diagnostic categories (B1-5)

Category	n	%
B1	10	3.7
B2	183	68.5
B3	11	4.1
B4	3	1.1
B5a	5	1.9
B5b	54	20.2
B5d	1	0.4
Total	267	100.0

Table 3 outlines the distribution of samples according to ER and PR status. There were 57 samples (24.7%) that were included in the ER and PR testing. There were 25 (9.4%) samples that were negative for both ER and PR, while 28 (10.5%) were positive for both ER and PR. There were four (1.5%) samples positive for ER and negative for PR. The association between ER and PR status was statistically significant with a p<0.001.

**Table 3:** Distribution of samples according to ER and PR status (N=267) *Non-diagnostic category includes mostly cases with insufficient tissue or when IHC stains didn't work properly **Not applicable (N/A) category includes all the non-malignant cases in categories (B1-B4) ER: estrogen receptor, PR: progesterone receptor

Distribution of samples according to ER and PR status		PR status							
		Negative		Positive		Non-diagnostic*		Not applicable (N/A)**	
		n	%	n	%	n	%	n	%
ER status	Negative	25	9.4%	1	0.4%	1	0.4%	2	0.7%
	Positive	4	1.5%	28	10.5%	1	0.4%	1	0.4%
	Non-diagnostic*	0	0.0%	1	0.4%	1	0.4%	1	0.4%
	Not applicable (N/A)**	0	0.0%	0	0.0%	0	0.0%	201	75.3%

**Figure 3:**
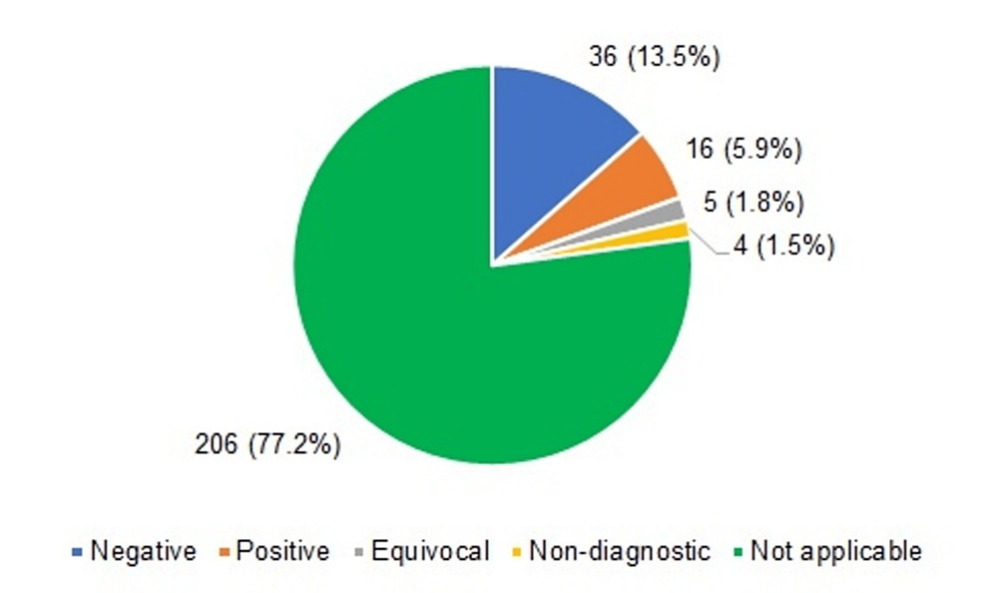
Pie chart showing the distribution of samples according to HER2 status

Both Table 4 and Figure 3 provide the distribution of samples according to HER2 status. There were 61 samples in which the test was performed. Out of these, maximum samples (36, 13.5%) were negative, while 16 (6.0%) were positive. There were five (1.9%) equivocal samples.

**Table 4:** Distribution of samples according to HER2 status *Non-diagnostic category includes mostly cases with insufficient tissue or when IHC stains didn’t work properly **Not applicable (N/A) category includes all the non-malignant cases in categories (B1-B4) as well as cases of in situ carcinoma (B5a) where HER2 testing is not indicated HER2: human epidermal growth factor receptor 2

HER2 status	n	%
Negative	36	13.5%
Equivocal	5	1.9%
Positive	16	6.0%
Non-diagnostic*	4	1.5%
Not applicable (N/A)**	206	77.2%
Total	267	100.0%

Table 5 provides the distribution of samples according to ER, PR, and HER2 status jointly. There were 16 (6.0%) samples that were ER, PR, and HER2 negative (triple negative), while eight (3.0%) samples were ER and PR negative but HER2 positive. There were 17 (6.3%) samples positive for ER and PR but negative for HER2.

**Table 5:** Distribution of samples according to HER2, ER, and PR status (N=267) *Non-diagnostic category includes mostly cases with insufficient tissue or when IHC stains didn’t work properly **Not applicable (N/A) category includes generally all the non-malignant cases in categories (B1-B4) HER2: human epidermal growth factor receptor 2, ER: estrogen receptor, PR: progesterone receptor

ER status	PR status	HER status	n (%)
Negative	Negative	Negative	16 (6.0)
		Positive	8 (3.0)
		Non-diagnostic*	1 (0.4)
	Positive	Negative	1 (0.4)
	Non-diagnostic*	Non-diagnostic*	1 (0.4)
	N/A**	N/A**	2 (0.7)
Positive	Negative	Negative	2 (0.7)
		Positive	2 (0.7)
	Positive	Equivocal	5 (1.9)
		Negative	17 (6.4)
		Positive	5 (1.9)
		N/A**	1 (0.4)
	Non-diagnostic*	Positive	1 (0.4)
	N/A**	N/A**	1 (0.4)
Non-diagnostic*	Positive	Non-diagnostic*	1 (0.4)
	Non-diagnostic*	Non-diagnostic*	1 (0.4)
	N/A**	N/A**	1 (0.4)
N/A**	N/A**	N/A**	201 (75.3)

**Figure 4:**
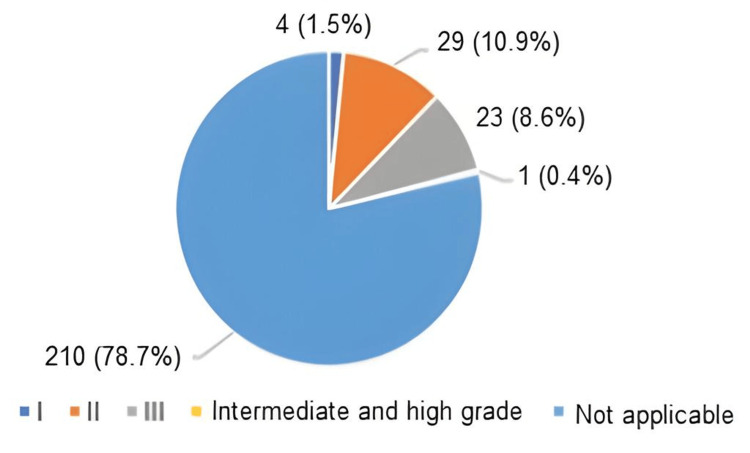
Pie chart showing the distribution of samples according to grade

Both Table 6 and Figure 4 provide the distribution of samples according to tumor grade. Out of the total 267 samples, grading was not done in 210 non-malignant samples from categories B1-B4 (78.7%). Out of the malignant samples, 29 (10.9%) were with grade II, followed by 23 (8.6%) with grade III and four (1.5%) with grade I.

**Table 6:** Distribution of samples according to tumor grade *Not applicable (N/A) category includes all the non-malignant cases in categories (B1-B4)

Grade	n	%
I	4	1.5
II	29	10.9
III	23	8.6
Intermediate and high grade	1	0.4
N/A*	210	78.7
Total	267	

Table 7 gives the cross-table showing the distribution of samples according to age and grade. Out of 267 samples, the grades were obtained for 57 samples. Maximum samples (14) were in the age range of 36-40 years and grade II, followed by 13 in the sample age range with grade III. There were four samples in the age range of 31-35 years with grade I. The association between age categories and grade was not statistically significant (p=0.113).

**Table 7:** Cross-table showing the distribution of samples according to age and grade *IHG: intermediate and high grade **Not applicable (N/A) category includes all the non-malignant cases in categories (B1-B4)

Distribution of samples according to age and grade		Grade category					Total
		I	II	III	IHG*	N/A**	
Age category	≤20	0	0	0	0	26	26
	21-25	0	2	1	0	33	36
	26-30	0	2	4	0	31	37
	31-35	4	11	5	1	57	78
	36-40	0	14	13	0	63	90
Total		4	29	23	1	210	267

Table 8 gives the cross-table showing the distribution of samples according to age and category. Maximum samples (53) were in the age range of 31-35 years with category B2, followed by 52 samples in the age range of 36-40 years with category B3, 29 in the age range of 26-30 years with category B2, 26 in the age range of 21-25 years with category B2, and 23 in the age range of ≤20 years with category B2. There were five samples in the age range of 21-25 years with category B3, while three samples were in the range of 31-35 years with category B3. Equally the same number of samples were in the age range of 36-40 years with category B1. The association between age and categories was statistically significant with a p-value of 0.0002. A significantly higher proportion of cases with B5a or B5b categories were in the age range of 31-35 and 36-40 years.

**Table 8:** Cross-table showing the distribution of samples according to age and category

Distribution of samples according to age and category		Category						
		B1	B2	B3	B4	B5a	B5b	B5d
Age category (years)	<=20	2	23	1	0	0	0	0
	21-25	2	26	5	0	0	3	0
	26-30	1	29	0	0	0	6	1
	31-35	2	53	3	0	1	19	0
	36-40	3	52	2	3	4	26	0
	Total	10	183	11	3	5	54	1

## Discussion

Over 25% of women may encounter breast diseases at some point in their lives, and the incidence of breast diseases increases with age [1]. However, not all breast masses are cancerous; depending on the patient’s age and hormonal balance, they may be benign or malignant. Triple assessment is the gold standard for evaluating breast masses. It is a multidisciplinary structured approach that involves clinical, radiological, and histopathological assessment [1]. Breast masses that occur in young females are usually benign in nature. These include masses due to mastitis and breast abscesses which are commonly caused by *Staphylococcus aureus* infection and are usually associated with lactation, fibroadenoma which is a mobile rubbery mass commonly seen in women under the age of 30, and pseudo-angiomatous stromal hyperplasia which is also commonly seen in premenopausal women [1,8]. Therefore, these findings were in accordance with our results since we identified that benign conditions are most seen in younger age groups.

Breast cancer is rare in women under the age of 40, making it more challenging to diagnose a malignant tumor since benign breast lumps predominate in this age category [9]. It is well known that the prognosis is poorer and the biological behavior is more aggressive in younger individuals. This is because of the triple-negative hormonal and HER2 receptor status, advanced presentation, higher differentiation grade, limited screening, lymphovascular invasion, and delayed diagnosis [10]. On the contrary, the comparison between age and grade in our study was not statistically significant in all age groups including the younger individuals.

In postmenopausal women, breast cancer is a common phenomenon, and it can be divided into invasive and non-invasive based on histopathological findings and can be treated based on their hormone receptor status (ER, PR, and HER2) [1]. In accordance with our study, it was identified that benign breast mass (B2 category) was the most common among women aged 40 and below with 183 samples (68.5%), followed by malignant breast mass (B5b category) with 54 samples (20.2%). In a study by Singh et al., benign breast lumps were commonly seen in the age group of 21-30 years followed by 11-20 years, which commonly presented with fibroadenoma [11]. Moreover, in relation to our study, 71 samples presented with benign fibroadenoma in the age group of 16-37 years. Our study outlines a comprehensive assessment of breast masses diagnosed by biopsies in women up to the age of 40 in Bahrain from January 2020 to January 2023. This study analyzed the distribution of samples based on age, category, hormone receptor (ER and PR) status, HER2 status, and grade. The distribution of samples ranged between 36 and 37 biopsies among women aged 21-30; however, the age group of 36-40 years had the highest number of biopsies accounting for 90 biopsies.

A thorough clinical assessment, including personal and family history and physical examination, is an essential step for identifying risk factors for malignancy. These include exposure to exogenous hormones, age at menarche, history of childbearing, and lifestyle-related risk factors [12]. However, a positive family history is a significant risk factor when it comes to breast cancer. Thus, it is crucial to obtain a complete and relevant history. Some patients may inherit genetic mutations that increase the risk of developing breast cancer, such as BRCA1, BRCA2, TP53, and PALB3, among others [12]. Identification of genetic mutations and understanding the clinical and pathological aspects are crucial for promoting tailored treatment approaches, frequent screening, and the need for prophylactic surgery (for the patient) and genetic counseling for the patient's first-degree relatives. Clinical examination involves assessing both breasts and axillae and, if indicated by history, other body systems as well [1].

Furthermore, radiological imaging is important and is not performed for the early detection of breast cancer but rather for high-risk or symptomatic patients. Ultrasonography is chosen as a modality to detect breast lumps and distinguish between benign and malignant ones. In comparison with mammography, breast density reduces the accuracy of mammography and its ability to detect microcalcifications [8]. Mammography or MRI is preferred in high-risk patients with inherited mutations or when malignancy is suspected [8]. The American College of Radiologists recommends using the BI-RADS for reporting breast lesions. It is used by radiologists to categorize radiological findings into degrees of suspicion for malignancy [13].

It is essential to establish good radiological and pathological concordance, and all dis-concordances should be reviewed in a specialized breast multi-disciplinary meeting.

The BI-RADS is a radiological risk assessment reporting scheme for breast lesions, utilizing ultrasound, mammography, and MRI. It facilitates an easy and accurate system of breast pathology communication with the referring physicians, which will outline the management [14,15].

It comprises six categories: BI-RADS 0 is an incomplete study that requires additional imaging. BI-RADS 1 is negative, with a 0% probability of malignancy. BI-RADS 2 are benign lesions with a 0% probability of malignancy. Lesions in this category include involuting or calcified fibroadenoma, vascular calcification, intramammary lymph nodes, and fat-containing lesions. Both BI-RADS 1 and 2 patients undergo routine screening. However, BI-RADA 3 has less than a 2% probability of malignancy and requires short-interval follow-up (around six months), such as focal asymmetry that becomes less dense on spot compression views. BIRAD 4 is suspicious of malignancy, with a probability ranging from 2% to 94%, and a biopsy is recommended. It is subdivided into three categories: 4A, low suspicion (2-10%, such as an abscess, complex cyst of a partially circumscribed mass); 4B, low suspicion (10-50%); and 4C, high suspicion (50-95%). Consequently, BI-RADS 5 is highly suggestive of malignancy, with a probability exceeding 95%, such as an ill-defined irregular, spiculated dense mass with architectural distortion. Lastly, BI-RADS 6 comprises biopsy-proven malignancy lesions [14-17].

Additionally, an ultrasound report should comment on the breast composition and provide a comprehensive description of the mass, including the shape, margin, orientation, echogenicity, presence of calcifications and their morphology, and other association features, such as architectural distortion, skin thickening and retraction, and mass vascularity [14-17]. Furthermore, a detailed mammogram report should also comment on the breast composition, mass (shape, margin, and density), asymmetry, architectural distortion, calcifications, and some other associated features, including lymphadenopathy [14-17]. Of note, amorphous, course heterogeneous, and fine pleomorphic calcifications are considered to be BI-RADS 4B, whereas fine linear or linear branching is considered to be BI-RADS 4C [14-17].

Finally, pathological analysis requires an invasive procedure to allow for histopathological assessment of the patient's breast mass. Diagnosis is usually made after an image-guided core needle biopsy (CNB) of the breast and then categorizing the mass based on the breast diagnostic categories in Table 9 [13]. To be able to interpret the results of any CNB, pathologists must have sufficient knowledge of the targeted radiological lesion [13].

**Table 9:** Breast diagnostic categories Adapted from Ellis et al. and Rahka et al. [13] ADH: atypical ductal hyperplasia, ALH: atypical lobular hyperplasia, LCIS: lobular carcinoma in situ, DCIS: ductal carcinoma in situ

Category	Significance	Included lesions
B1	Normal tissue	Lipomas, hamartomas, lactational change, excessive crush artifacts, bloody specimens
B2	Benign lesion	Fibroadenoma, fibrocystic change, sclerosing adenosis, ductal ectasia, breast inflammatory pathology, benign skin lesions, usual hyperplasia, columnar changes without atypia, papillomas without atypia <2 mm
B3a	Uncertain malignant potential without atypia	Fibroepithelial lesion with cellular stroma, radial scar, papillary lesions without atypia, adenomyoepithelioma, mucocele-like lesions without atypia, nipple adenomas, microglandular adenosis, granular cell tumor, spindle cell lesions
B3b	Uncertain malignant potential with atypia	ADH, ALH, classic LCIS, papillary lesions with atypia, atypical microglandular adenosis, flat epithelial atypia, radial scar or mucocele-like lesions with atypia
B4	Suspicious of malignancy	Cores that probably contain a carcinoma but it is not unequivocal in the sample
B5a	In situ carcinoma	DCIS, pleomorphic or florid LCIS
B5b	Invasive carcinoma	Invasive carcinoma, lymphomas, sarcomas, metastases to the breast
B5c	Carcinoma, not possible to differentiate invasive or in situ	Very unusual fragments of malignant epithelia without stroma

This is done after considering the patient's risk-benefit and the suspicious lump detected during the previous two assessments [1]. In this study, all samples were collected using different methods of attaining breast mass biopsies, which are considered the gold standard for diagnosing breast lumps. IHC stains are usually performed to assess ER, PR, and HER2 expressions. This will help categorize the breast masses into specific categories and determine the appropriate treatment [8].

The RCPath guidelines for non-operative diagnostic procedures and reporting in breast cancer screening recommend categorizing the pathological findings into five diagnostic categories. These categories only take histological characteristics into account. The histopathological B classification of CNB of the breast is classified as B1 (not diagnostic/normal tissue), B2 (benign), B3 (lesions with uncertain potential), B4 (suspicious of malignancy), and B5 (malignant), as shown in Table 9 [13].

B3 are lesions of uncertain malignant potential, and these have been the area of interest in recent years. They represent a wide range of histopathological entities with variable risk of associated malignancy; hence, there are no definitive guidelines and consensus on how they should be managed. These include fibroepithelial lesions, papilloma, radial scars/complex sclerosing lesions, atypical ductal hyperplasia, flat epithelial atypia, atypical lobular hyperplasia, and lobular carcinoma in situ. Although these lesions on their own may not necessarily be malignant, malignant change can sometimes coexist with the lesion. Therefore, the presence of the lesion itself increases the risk of subsequent breast malignancy over time [18-20].

Historically, all B3 lesions were managed with surgical excision, regardless of the underlying lesion type. It is essential to identify any co-existing malignancies. However, this would mean unnecessary surgery for a large proportion of women. The National Institute for Health and Care Excellence guidelines suggested that there is sufficient evidence regarding the safety and efficacy of image-guided large-volume biopsies such as vacuum-assisted biopsy for B3 lesions [19]. However, this risk of missing the area of malignancy with a B3 lesion varies depending on the underlying B3 lesion subgroup, with some lesions having a lower risk of missed malignancy than others. A B3 lesions' risk of malignancy can fluctuate from 0% to 79%, which makes developing a solid management strategy difficult due to the fluctuating risk of malignancy [21]. Therefore, it is recommended to discuss all patients with B3 diagnoses in a multidisciplinary breast meeting before deciding on clinical management to ensure radiological and clinical correlation.

B4 lesions are the least common (<1%), and in most cases, either an excisional biopsy or a new CNB is advised to obtain a definitive diagnosis [22,23].

For all invasive carcinomas (B5), our reporting system follows the recommendation of the College of American Pathologists breast invasive biopsy cancer protocol which includes essential components such as grade, histological subtype, and assessment of prognostic and predictive factors such as Ki67, ER, and HER2 assessment. New developments, including assessments of the HER2-low category, are currently being incorporated into standard clinical practice; although retrospective review of the HER2-low diagnostic category is not currently being mandated, it is being done on a case-by-case basis. All cases of invasive malignancy are usually discussed in breast multidisciplinary team meetings to decide if patients are candidates for neoadjuvant treatment or upfront surgery.

There are a few limitations to be considered in the current study. The sample size of the breast mass samples was limited due to the fact that they were taken from a single center in the Kingdom of Bahrain. In addition, the specified time frame was limited and took place during the COVID-19 pandemic, which may affect and limit the samples’ exposure. In order to validate our results, a larger clinical trial would be necessary. Furthermore, due to a lack of research papers relevant to our subject matter in Bahrain, our interpretations and comparisons are limited. Our study precisely included a single center, which may or may not be generalizable to all women in Bahrain.

## Conclusions

The purpose of this study was to analyze the frequency and incidence of breast mass specimens among women up to the age of 40 in Bahrain in the past three years (2020-2023). Our study included age, histopathological characteristics, radiological findings, and hormonal status which were the parameters used to compare models. The results revealed that the incidence of breast masses in women aged 30 and above was significantly higher than that of younger individuals, thus demonstrating a possible increased likelihood of developing breast diseases with an increase in age. Moreover, malignancy rates were observed to be higher among women over the age of 30. Further extensive studies are recommended to involve a wider range of samples to have precise data, as well as to use samples from other tertiary hospitals in Bahrain. This will help to increase the generalizability of the findings and provide a more comprehensive understanding of breast malignancies in women aged 40 and below.
